# Secretagogin expression in the vertebrate brainstem with focus on the noradrenergic system and implications for Alzheimer’s disease

**DOI:** 10.1007/s00429-019-01886-w

**Published:** 2019-05-29

**Authors:** Péter Zahola, János Hanics, Anna Pintér, Zoltán Máté, Anna Gáspárdy, Zsófia Hevesi, Diego Echevarria, Csaba Adori, Swapnali Barde, Beáta Törőcsik, Ferenc Erdélyi, Gábor Szabó, Ludwig Wagner, Gabor G. Kovacs, Tomas Hökfelt, Tibor Harkany, Alán Alpár

**Affiliations:** 1 SE NAP B Research Group of Experimental Neuroanatomy and Developmental Biology, Semmelweis University, Budapest, Hungary; 2 Department of Anatomy, Semmelweis University, Budapest, Hungary; 3 Institute of Experimental Medicine, Hungarian Academy of Sciences, Budapest, Hungary; 4 Department of Molecular Neurosciences, Center for Brain Research, Medical University of Vienna, 1090 Vienna, Austria; 5 Institute of Neuroscience, University of Miguel Hernandez de Elche, Alicante, Spain; 6 Department of Neuroscience, Karolinska Institutet, Biomedicum 7D, SE-17165 Stockholm, Sweden; 7 Department of Medical Biochemistry, Semmelweis University, Budapest, Hungary; 8 Department of Internal Medicine III, Medical University of Vienna, Vienna, Austria; 9 Institute of Neurology, Medical University of Vienna, Vienna, Austria

**Keywords:** Alzheimer’s disease, Calcium-binding proteins, Locus coeruleus, Norepinephrine, Phylogenetic conservation

## Abstract

Calcium-binding proteins are widely used to distinguish neuronal subsets in the brain. This study focuses on secretagogin, an EF-hand calcium sensor, to identify distinct neuronal populations in the brainstem of several vertebrate species. By using neural tube whole mounts of mouse embryos, we show that secretagogin is already expressed during the early ontogeny of brainstem noradrenaline cells. In adults, secretagogin-expressing neurons typically populate relay centres of special senses and vegetative regulatory centres of the medulla oblongata, pons and midbrain. Notably, secretagogin expression overlapped with the brainstem column of noradrenergic cell bodies, including the locus coeruleus (A6) and the A1, A5 and A7 fields. Secretagogin expression in avian, mouse, rat and human samples showed quasi-equivalent patterns, suggesting conservation throughout vertebrate phylogeny. We found reduced secretagogin expression in locus coeruleus from subjects with Alzheimer’s disease, and this reduction paralleled the loss of tyrosine hydroxylase, the enzyme rate limiting noradrenaline synthesis. Residual secretagogin immunoreactivity was confined to small submembrane domains associated with initial aberrant tau phosphorylation. In conclusion, we provide evidence that secretagogin is a useful marker to distinguish neuronal subsets in the brainstem, conserved throughout several species, and its altered expression may reflect cellular dysfunction of locus coeruleus neurons in Alzheimer’s disease.

**Electronic supplementary material:**

The online version of this article (10.1007/s00429-019-01886-w) contains supplementary material, which is available to authorized users.

## Introduction

**Fig. 1:**
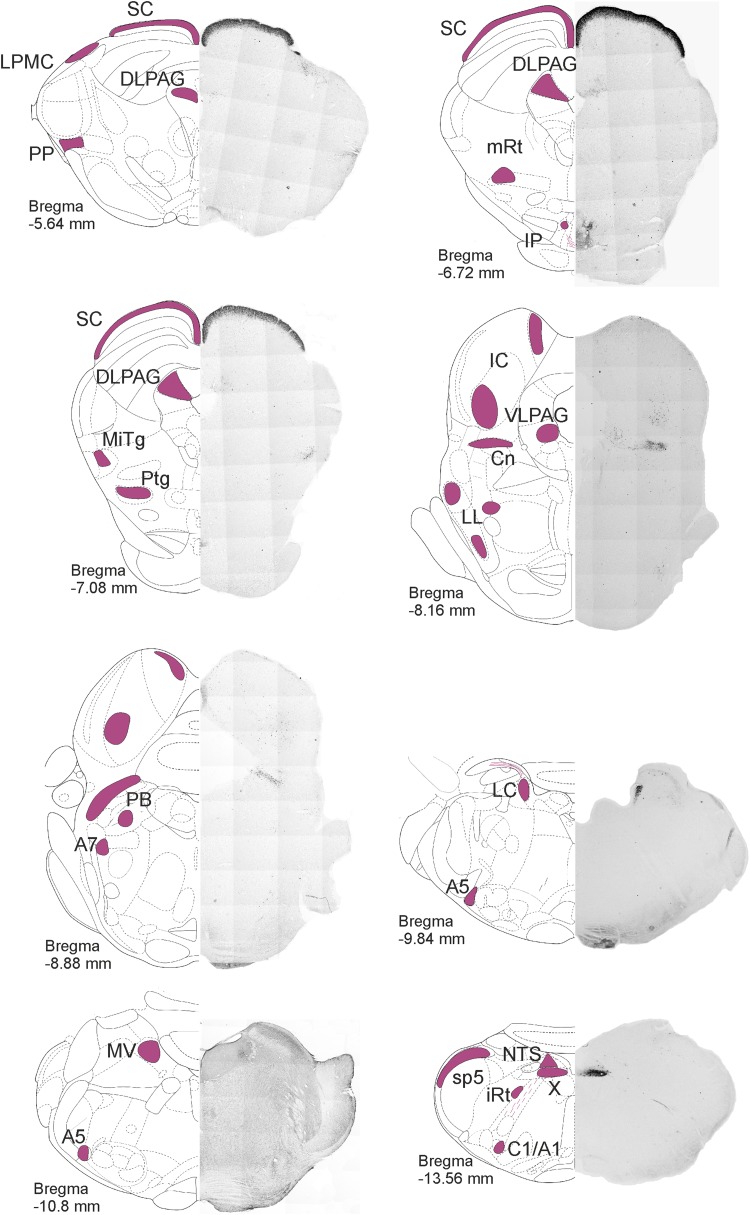
Distribution of secretagogin in the rat brainstem I. Secretagogin^+^ somata were identified in coronal sections throughout the rat brainstem. Low-power micrographs are paired with schemata of coronal brain sections to indicate craniocaudal levels. Secretagogin^+^ neurons-containing regions are indicated in purple. Superior colliculus, the microcellular tegmental nucleus, the dorsal nucleus of vagus and the noradrenergic cell groups including A1, A5, A6 and A7 typically expressed secretagogin. For high-power images, please see Fig. 4. *A1, A5, A7* noradrenergic cell groups, *C1* adrenergic cell group, *Cn* cuneiform nucleus, *DLPAG* dorsolateral periaqueductal grey, *IC* inferior colliculus, *IP* interpeduncular nucleus, *iRT* intermediate reticular nucleus, *LC* locus coeruleus, *LL* lateral lemniscus, *LPMC* lateral posterior thalamic nucleus mediocaudal part, *MiTg* microcellular tegmental nucleus, *mRT* mesencephalic reticular formation, *MV* medial vestibular nucleus, *NTS* solitary tract nucleus, *PB* parabrachial nucleus, *PP* peripeduncular nucleus, *Ptg* pedunculopontine tegmental nucleus, *sB* subbrachial nucleus, *SC* superior colliculus, *sp5* spinal tract nucleus of V, *VLPAG* ventrolateral periaqueductal grey, *X* dorsal nucleus of X

Our brainstem harbours a wealth of distinctly or ambiguously demarcated cell groups with various functions serving basic physiological needs. Pathways of special senses have relay centres in the cranial brainstem: the superior colliculus and the lateral geniculate nucleus of the visual system, and the inferior colliculus and the medial geniculate nucleus of the acoustic system are situated in the mesencephalon. Cranial nerve nuclei of different modalities are arranged in logical mediolateral and craniocaudal orders, reaching from the caudal medulla oblongata to the cranial midbrain. These include stations of specific sensory pathways, like the cochlear and vestibular nuclei in the caudal pons, or complex vegetative centres, like the solitary tract nucleus in the medulla oblongata. Other autonomic integrative centres, like the periaqueductal grey involved in behavioural responses to threatening stimuli (Faull et al. 2019) and opioid modulation of pain (Martins and Tavares 2017), or the parabrachial nuclear complex controlling fluid and food homoeostasis, cardiovascular functions (Davern 2014) and body temperature (Morrison and Nakamura 2011) appear as independent nuclei in the brainstem. Activating systems using biogenic amines as neurotransmitters have their origin in the brainstem: noradrenaline- (A1–A6 fields), adrenaline- (C1–C3 fields) or serotonin-containing (B1–B8 fields) neurons from nuclei, and their projections richly innervate many forebrain regions, the cerebellum and the spinal cord. The nuclei and centres of these different systems (Fig. 1) are intertwined and may in the brainstem even overlap, which has made their identification and separation an ongoing challenge.

The classical neuroanatomical toolbox including, e.g., ubiquitous neuronal staining or metal-based impregnations, was the first pioneering step to identify major brainstem centres. The advent of tract tracing and immunohistochemistry opened a new dimension in understanding the layout of brainstem circuits. Researchers not only identified hitherto unknown nuclei, but were able to demarcate subregions, further unravelling cellular heterogeneity within cell groups previously thought to be homogenous (Dahlstroem and Fuxe 1964; Ljungdahl et al. 1978; Markia et al. 2008; Palkovits 1999; Zaborszky et al. 1984). Although chemogenetic and optogenetic tools brought a quantum leap in the functional classification of neurons, neurochemical markers remain of foremost importance to identify neuronal populations in the brain (Rees et al. 2017), with calcium-binding proteins (CaBPs) being common-choice candidates to distinguish cell types or nuclei (Andressen et al. 1993; Freund and Buzsáki 1996; Riedel et al. 2002).

Activation of signalling pathways is a typical calcium (Ca^2+^)-dependent mechanism with Ca^2+^-sensor proteins specifying downstream protein–protein interactions (Skelton et al. 1994). Secretagogin, a Ca^2+^-sensor protein (Wagner et al. 2000), undergoes conformational changes upon Ca^2+^ binding (Rogstam et al. 2007) to primarily affect protein turnover and exocytosis (Gartner et al. 2007). In agreement with its involvement in the exocytotic machinery, secretagogin, found in the mammalian rodent and non-primate (Hanics et al. 2017; Mulder et al. 2009, 2010) and human (Attems et al. 2012a) brain and spinal cord (Zhang et al. 2016), has been associated with presynaptic neurotransmitter release (Romanov et al. 2015; Zhang et al. 2016). Nevertheless, while forebrain distribution of secretagogin-expressing^(+)^ neurons is well characterized (Alpar et al. 2012; Attems et al. 2008; Garas et al. 2016; Gyengesi et al. 2013; Kosaka and Kosaka 2013; Kosaka et al. 2017; Mulder et al. 2009; Romanov et al. 2015), the localization and phenotype of secretagogin^+^ neurons in the brainstem remain elusive.

Here, we use secretagogin as a valuable neuroanatomical marker to identify brainstem nuclei in vertebrates, including avian, rodent and human brains. In addition to relay centres of special senses and vegetative regulatory centres, we classify the brainstem noradrenaline stress axis as a focus of secretagogin expression in the murine and human brain. Noradrenergic neurons, assembled in a column of nuclei throughout the medullary and pontine brainstem (Dahlstroem and Fuxe 1964), are extensively connected to cortical and subcortical forebrain regions to orchestrate central responses to, e.g. stress (Aston-Jones et al. 1996; Itoi and Sugimoto 2010; Samuels and Szabadi 2008a, b). Imbalance in noradrenaline function may result in affective, panic and anxiety disorders (Bremner et al. 1996a, b; Charney 2003; Kvetnansky et al. 2009; Samuels and Szabadi 2008a, b), representing serious and costly morbidities and a burden on public health worldwide (Alloul et al. 1998; Takizawa et al. 2015). Of note, locus coeruleus is critically vulnerable already in the initial phase of neurodegenerative diseases, most notably Alzheimer’s disease (Braak and Del Tredici 2012; Tomlinson et al. 1981). We suggest that altered secretagogin expression in locus coeruleus neurons is a clinicopathological sign of Alzheimer’s disease paralleling or even preceding tyrosine hydroxylase (TH) loss.

## Results

### Secretagogin is expressed in the locus coeruleus of the early rhombencephalic neural tube

**Fig. 2:**
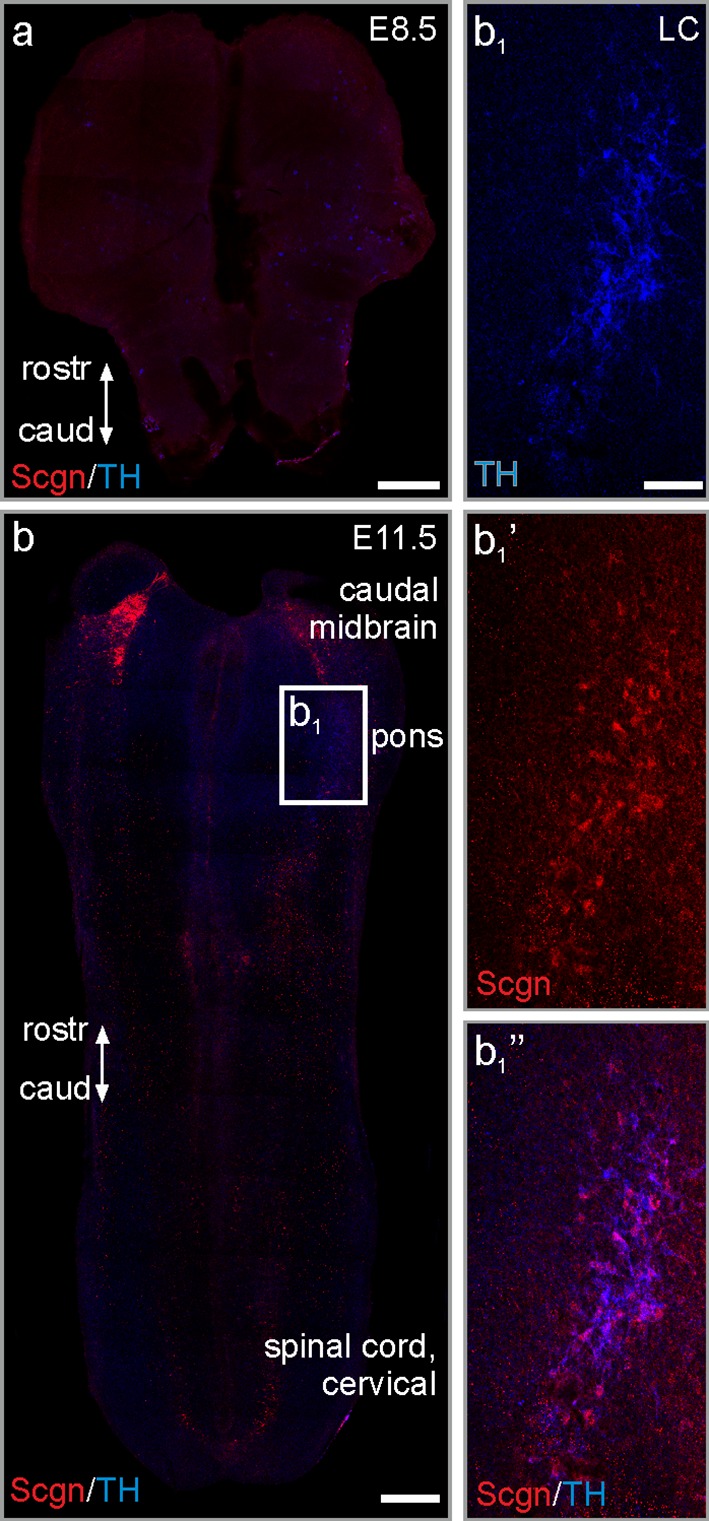
Secretagogin expression in the early neural tube. **a** Neural tube whole mount from E8.5 mouse embryos lacks secretagogin immunoreactivity. **b** Secretagogin expression appears at E11.5 in the caudal midbrain. **b**_**1**_–**b**_**1**_**’’** TH^+^ neurons share secretagogin immunoreactivity in the locus coeruleus. *caud* caudal, *rostr* rostral, *Scgn* secretagogin, TH tyrosine hydroxylase. Scale bars 300 μm (**a**, **b**), 60 μm (**b**_**1**_)

We have isolated and prepared neural tube whole mounts from early mouse embryos to determine the onset of secretagogin expression in the foetal brainstem (Fig. 2). At embryonic day 8.5 (E8.5), no secretagogin immunoreactivity could be detected in any rhombencephalic domain (Fig. 2a). Brainstem neurons began to express secretagogin by E11.5, with a “hot spot” in the caudal midbrain (Fig. 2b) and in cell contingents along the basal plate and limiting sulcus of the medulla oblongata and the cervical flexure. At E11.5, TH expression was detected in a cell population dispersed throughout the medulla oblongata and pons. Those TH^+^ cells that had concentrated in the locus coeruleus (Fig. 2b, b_1_) were secretagogin immunoreactive (Fig. 2b_1_’, b_1_’’) with TH^+^/secretagogin^−^ cells being the exception rather than the rule. We conclude that secretagogin and TH are expressed coincidentally in the major noradrenergic brainstem centre during mouse ontogeny.

### Distribution of secretagogin^+^ neurons in the rodent brainstem

**Fig. 3:**
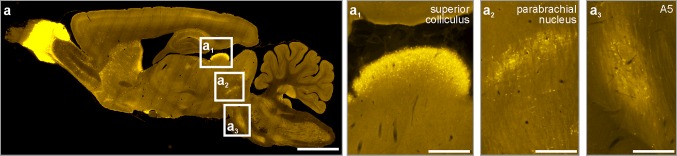
Distribution of secretagogin in the rat brainstem II. Sagittal sections of the rat brain revealed superior colliculus (**a**_**1**_), the parabrachial nucleus (**a**_**2**_) and the A5 field (**a**_**3**_) as typical loci which harbour secretagogin^+^ neurons. Scale bars 1 mm (**a**), 300 µm (**a**_**1**_, **a**_**2**_, **a**_**3**_)

**Fig. 4:**
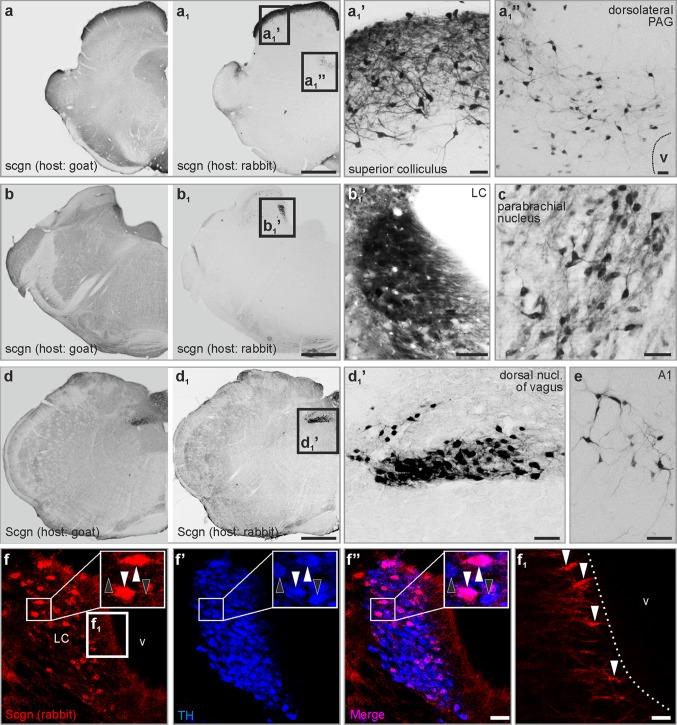
Distribution of secretagogin in the rat brainstem III. (**a**–**a**_**1**_**’’**) In the midbrain, secretagogin^+^ neurons condensed in the superior colliculus and the dorsolateral periaqueductal grey. **b**–**c** Pontine distribution of secretagogin expression. Locus coeruleus, and lateral to it the parabrachial nucleus (**c**) contained many secretagogin^+^ cells. (**d**–**d**_**1**_**’**) In the medulla oblongata, the dorsal nucleus of vagus was clearly outlined by its content of secretagogin^+^ neurons. **e** Secretagogin^+^ cells in the A1 field. **f**–**f’’** Secretagogin was typically expressed in TH^+^ neurons in the locus coeruleus (white arrowheads indicate TH^+^/secretagogin^+^ neurons, black arrowheads point to TH^+^/secretagogin^−^ neurons). **f**_**1**_ The processes of secretagogin^+^ locus coeruleus neurons reached the ventricular surface (white arrowheads). *LC* locus coeruleus, *scgn* secretagogin, *PAG* periaqueductal grey. Scale bars 1 mm (**a**_**1**_, **b**_**1**_, **d**_**1**_), 100 µm (**a**_**1**_**’**), 40 µm (**a**_**1**_, **b**_**1**_**’**), 30 µm (**c**, **d**_**1**_**’**, **e**, **f’’**), 10 µm (**a**_**1**_**’’**), 3 µm (**f**_**1**_)

Our systematic survey through the rat brainstem revealed several medullary, pontine and midbrain domains which harboured secretagogin^+^ neurons (Fig. 1). In sagittal sections, i.a. the superior colliculus, the parabrachial nucleus and the A5 field emerged as secretagogin ‘hot spots’ (Fig. 3). Using serial coronal sections of the brainstem (Fig. 1), we identified secretagogin immunoreactivity in the superior colliculus and in the dorsolateral part of the periaqueductal grey in the midbrain (Fig. 4a–a_1_’’), the locus coeruleus, parabrachial nucleus in the pons (Fig. 4b, c), and the dorsal nucleus of vagus in the medulla oblongata (Fig. 4d_1_, d_1_’). We identified identical distributions of immunoreactive cells when using our primary antibodies produced either in rabbit or goat, although the goat antiserum was associated with higher tissue background (Fig. 4a, a_1_, b, b_1_, d, d_1_). In addition, several smaller groups of secretagogin^+^ neurons appeared in the medulla oblongata and pons, which were identified as the A1 (Fig. 4e), A5 and A7 fields. Secretagogin/TH double immunolabelling in the locus coeruleus (Fig. 4f–f’’) showed that 75.5 ± 6.9% (average ± SEM) of all labelled cells co-expressed these markers, leaving 23.3 ± 2.2% and 1.1 ± 0.9% of cells single labelled for TH and secretagogin, respectively. The processes of secretagogin^+^ locus coeruleus neurons were typically directed towards the ventricular space, likely representing dendrites (Fig. 4f_1_). At the same time, TH^+^ neurons in the ventral tegmentum of the midbrain did not co-express secretagogin (Fig. S1a–a’’). Likewise, serotonin^+^ median raphe neurons were also immunonegative for secretagogin (Fig. S1b–b’’). Furthermore, in the ventral part of the periaqueductal grey, where serotonin^+^ and secretagogin^+^ neurons coincidentally occurred, we could not identify their co-expression either (Fig. S1c–c’’).

### Relation of secretagogin to other CaBPs

**Fig. 5:**
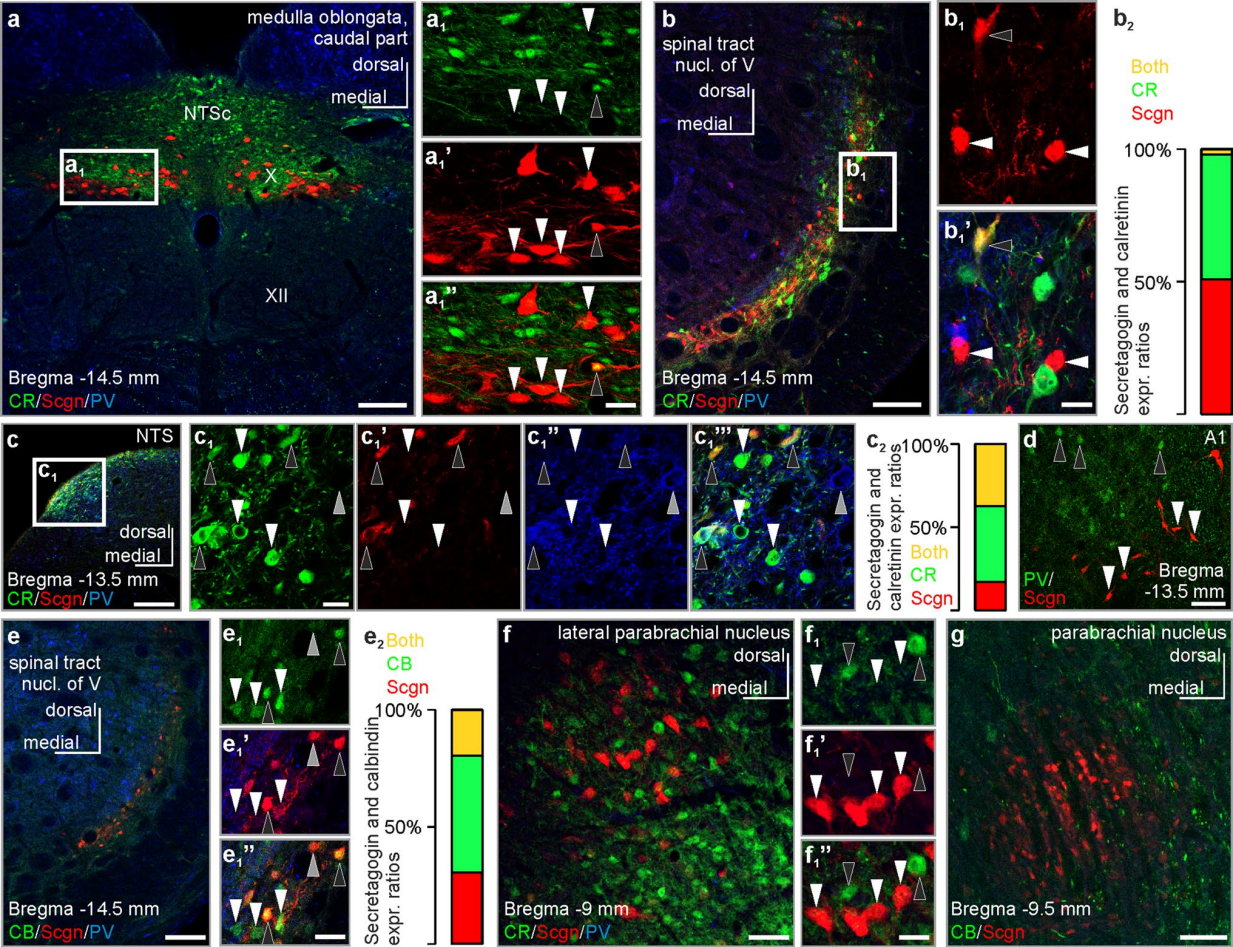
Co-expression of secretagogin with classical Ca^2+^-binding proteins in select brainstem nuclei of the rat. **a**–**a**_**1**_**’’** Complementary distribution of calretinin and secretagogin in the commissural part of the solitary nucleus and the dorsal nucleus of vagus (white arrowheads indicate secretagogin^+^/calretinin^−^ neurons, black arrowhead points to a secretagogin^+^/calretinin^+^ neuron). **b**–**b**_**1**_**’** Secretagogin^+^ neurons typically remained calretinin immunonegative (white arrowheads) in the spinal tract nucleus of the trigeminus, with exceptional co-expression only (black arrowhead). **b**_**2**_ Single- and co-expression ratios of secretagogin and calretinin in the spinal tract nucleus of the trigeminus. 100% percentage refers to all labelled cells detected for secretagogin and/or calretinin immunoreactivity. Secretagogin^+^/calretinin^+^ neurons: 2.8 ± 3.2%, secretagogin^+^/calretinin^−^ neurons 51.2 ± 6.1%, secretagogin^−^/calretinin^+^ neurons 47.5 ± 5.4% (as of average ± s.e.m.). **c**–**c**_**1**_**’’’** Calretinin^+^ neurons in the dorsolateral part of the solitary tract nucleus can either remain immunonegative for secretagogin (white arrowheads) or co-express it (black arrowheads) Grey arrowhead points to a CR^−^/Scgn^−^/PV^+^ neuron. **c**_**2**_ Single- and co-expression ratios of secretagogin and calretinin in the dorsolateral part of the solitary tract nucleus. 100% percentage refers to all labelled cells detected for secretagogin and/or calretinin immunoreactivity. Secretagogin^+^/calretinin^+^ neurons: 36.1 ± 2.3%, secretagogin^+^/calretinin^−^ neurons 16.9 ± 3.5%, secretagogin^−^/calretinin^+^ neurons 43.7 ± 2.1% (as of average ± s.e.m.). **d** Secretagogin^+^ neurons (white arrowheads) in the A1 field did not co-express parvalbumin (black arrowheads point to secretagogin^−^/parvalbumin^+^ somata). **e**–**e**_**1**_**’’** Secretagogin^+^ neurons typically co-expressed calbindin in the spinal tract nucleus of the trigeminus (black arrowheads). White arrowheads point to calbindin^+^/secretagogin^−^ somata, grey arrowhead to a secretagogin^+^/calbindin^−^ soma. **e**_**2**_ Single- and co-expression ratios of secretagogin and calbindin in the spinal tract nucleus of the trigeminus. 100% percentage refers to all labelled cells detected for secretagogin and/or calbindin immunoreactivity. Secretagogin^+^/calbindin^+^ neurons: 19.1 ± 8.0%, secretagogin^+^/calbindin^−^ neurons 30.7 ± 11.3%, secretagogin^−^/calbindin^+^ neurons 50.2 ± 7.2% (as of average ± s.e.m.). **f**–**f**_**1**_**’’** Secretagogin^+^ neurons showed a complementary distribution to calretinin^+^ neurons in the lateral parabrachial nucleus (white arrowheads point to secretagogin^+^/calretinin^−^ somata, black arrowheads point to secretagogin^−^/calretinin^+^ somata). **g** Secretagogin^+^ neurons remain immunonegative for calbindin in the parabrachial nucleus. *CB* calbindin, *CR* calretinin, *NTS* solitary tract nucleus, *NTSc* commissural part of the solitary tract nucleus, *Scgn* secretagogin, *PV* parvalbumin, *X* dorsal nucleus of vagus, *XII* hypoglossal nucleus. Scale bars 150 µm (**a**, **b**, **c**, **e**), 70 µm (**g**), 40 µm (**d**, **f**), 10 µm (**a**_**1**_**’’**, **b**_**1**_**’**, **c**_**1**_, **e**_**1**_**’’**, **f**_**1**_**’’**)

CaBPs are routinely used to typify neurons in the brain (Skelton et al. 1994). They exhibit largely non-overlapping expression patterns (Andressen et al. 1993; Freund and Buzsáki 1996) with occasional co-localization in the olfactory circuit, ventral pallidum and renewing cells of the dentate gyrus (del Rio and DeFelipe 1997; Wouterlood et al. 2001). In the brainstem, secretagogin^+^ neurons form largely non-overlapping populations with only occasional, domain-specific overlap/co-expression of CaBPs in select nuclei (Fig. 5): in the dorsal nucleus of vagus and commissural part of the solitary tract nucleus, a complementary expression pattern of calretinin and secretagogin was shown (Fig. 5a–a_1_’’), with many calretinin^+^/secretagogin^+^ double-labelled neurons in the dorsolateral part of the solitary tract nucleus at the same level (Fig. 5c–c_1_’’’). By using simultaneous labelling for secretagogin/calretinin/parvalbumin, 68.5 ± 5.4% of all secretagogin^+^ neurons co-expressed calretinin. Secretagogin/parvalbumin co-expression was exceptionally rare: we only identified a single secretagogin^+^ neuron which also harboured parvalbumin (Fig. 5c_1_’, c_1_’’). We found a diverse co-expression pattern in the spinal tract nucleus of the trigeminus: using secretagogin/calretinin/parvalbumin or secretagogin/calbindin D28k/parvalbumin immunohistochemistry, secretagogin often co-localized with calbindin D28k (Fig. 5e–e_2_, 40.3 ± 15.9% of all secretagogin^+^ neurons co-expressed calbindin D28k), only sporadically with calretinin (Fig. 5b–b_2_, 2.8 ± 3.2% of all secretagogin^+^ neurons co-expressed calretinin), but not with parvalbumin (Fig. 5b–b_1_’’, e–e_2_). Similarly, secretagogin^+^ neurons were immunonegative for parvalbumin in the ventrolateral medulla (Fig. 5d), as well as in the parabrachial nucleus (Fig. 5f). Secretagogin^+^ neurons in the parabrachial nucleus did not share calretinin (Fig. 5f–f_1_’’) or calbindin immunoreactivity (Fig. 5g), either. We acknowledge that the above proportions were calculated using an investigative method and not by stereology; thus, it provides approximate instead of accurate results regarding cell numbers/proportions.

### Secretagogin in the mouse and avian brainstem

**Fig. 6:**
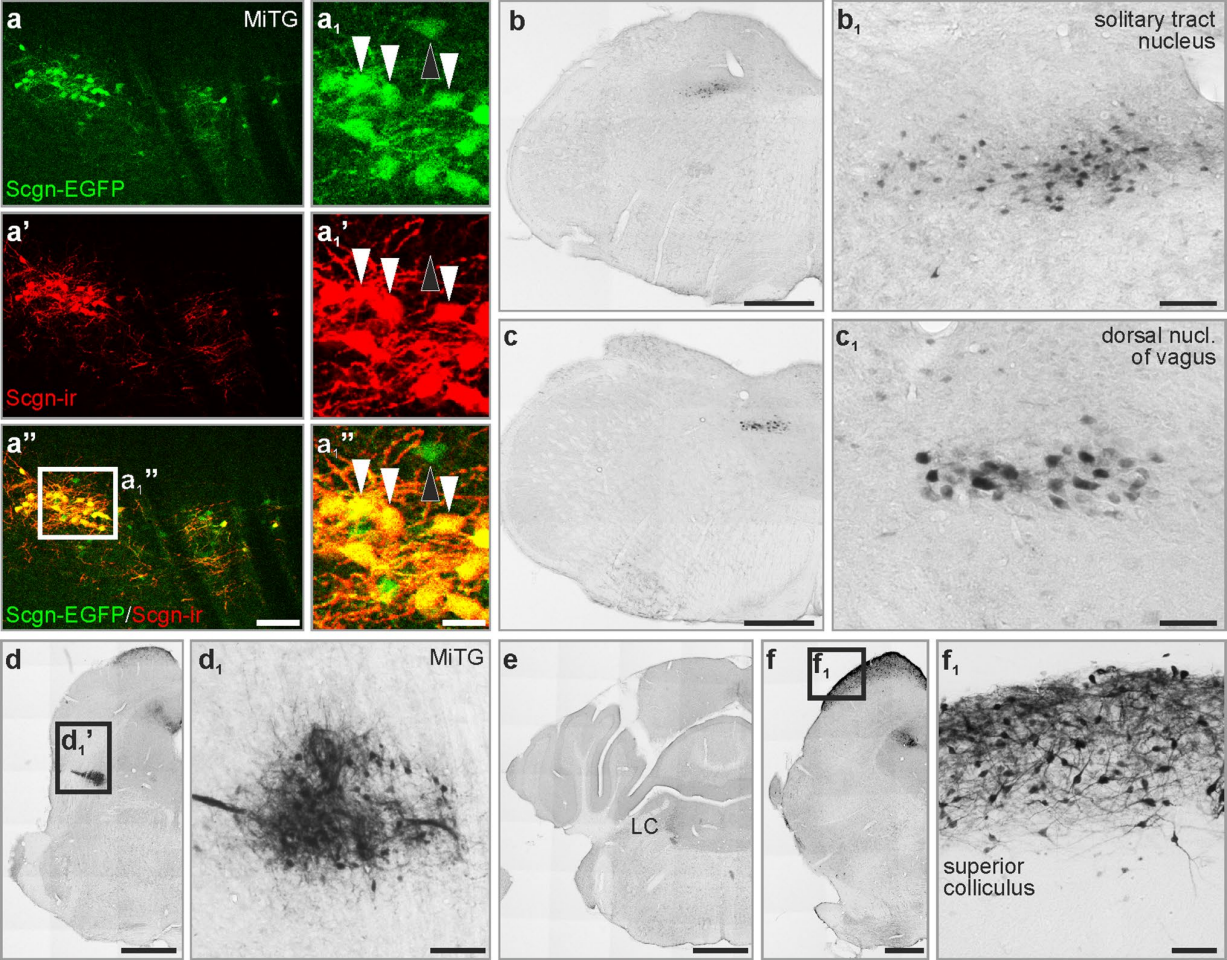
Secretagogin expression in the mouse brain stem. **a**–**a**_**1**_**’’** EGFP-expressing neurons in secretagogin–EGFP mice showed secretagogin immunoreactivity as exemplified in the microcellular tegmental nucleus (white arrowheads in **a**–**a**_**1**_**’’**). Occasionally, EGFP-expressing neurons remained secretagogin immunonegative (black arrowhead in **a**–**a**_**1**_**’’**). **b**–**f**_**1**_ Secretagogin^+^ neurons in the solitary tract nucleus (**b**, **b**_**1**_), dorsal nucleus of vagus (**c**, **c**_**1**_), microcellular tegmental nucleus (**d**, **d**_**1**_), locus coeruleus (**e**) and the superior colliculus (**f**, **f**_**1**_). *LC* locus coeruleus, *MiTg* microcellular tegmental nucleus. Scale bars 1 mm (**b**–**f**), 100 µm (**a’’**, **d**_**1**_, **f**_**1**_), 70 µm (**b**_**1**_), 40 µm (**c**_**1**_)

We next asked if secretagogin expression exhibited quasi-equivalent distribution in different vertebrates. To this end, serial sections of the mouse, chick and human brainstem were analysed for secretagogin^+^ neurons to see if an evolutionary continuum may exist among vertebrates. First, we generated a mouse line which expressed regulatory elements of the secretagogin promoter fused with EGFP on an artificial bacterial chromosome (*Scgn*^BAC/egfp^) to control for and validate our immunoreagents. We found extensive overlap between EGFP expression and secretagogin immunoreactivity (Fig. 6a–a_1_’’, Fig. S2) in brainstem nuclei (Table S1). The lack of secretagogin immunoreactivity in EGFP^+^ cells was attributed to low protein abundance in EGFP^+^ neurons, i.e. below immunocytochemical detectability (in our hands), because there was no hindbrain area in which the complete segregation of EGFP, suggesting ectopic expression, was seen. The distribution pattern of immunoreactive cells was largely similar to what we found in rats: secretagogin^+^ neurons typically occurred in the microcellular tegmental nucleus (Fig. 6a–a_1_’’, d, d_1_), nucleus of the solitary tract (6b, b_1_), the dorsal nucleus of vagus (Fig. 6c, c_1_), the noradrenergic fields, especially locus coeruleus (Fig. 6e), and the superior colliculus (Fig. 6f, f_1_). Yet, we detected differences between the two species: in the dorsolateral part of the periaqueductal grey, where immunoreactive somata were found in rat but only immunoreactive fibres and terminals in the mouse brain (Fig. S3a, a’). In rats, the interpeduncular nucleus contained secretagogin^+^ cell bodies in its dorsal versus secretagogin^+^ cell bodies and fibres in its lateral division: no such immunoreactivity was detected in the corresponding region of mice (Fig. S3b, b’). Similarly, no immunoreactivity was detected in the inferior colliculus (Fig. S3c, c’) and medial vestibular nucleus (Fig. S3d, d’) of mice.

### Secretagogin in the domestic chicken

**Fig. 7:**
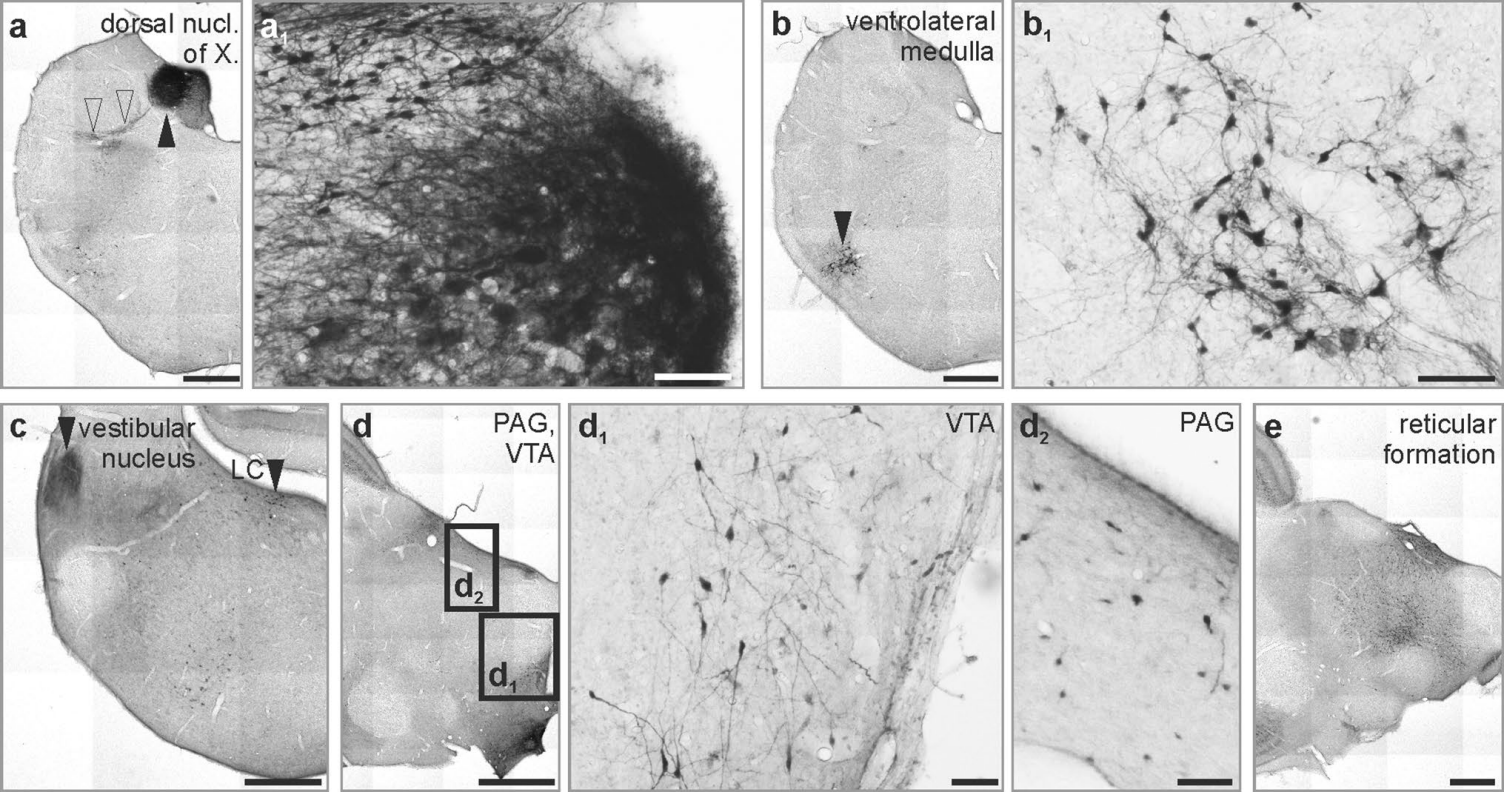
Secretagogin expression in the chicken brain stem. **a**–**e** The distribution of secretagogin^+^ neurons in the avian brain was similar to what we found in the mammalian brain. **a**–**b**_**1**_ In the medulla oblongata, immunoreactive neurons populated the dorsal nucleus of vagus (black arrowhead in a, open arrowheads indicate nerve fibres emanating from the nucleus) and the ventrolateral medulla (**b**, **b**_**1**_). **c** In the pons, immunoreactivity was typically confined to the vestibular area and the locus coeruleus. **d**–**e** In the midbrain, neurons of the periaqueductal grey matter, of the ventral tegmental area and scattered cells in the reticular formation showed secretagogin immunoreactivity. *LC* locus coeruleus, *PAG* periaqueductal grey, *VTA* ventral tegmental area. Scale bars 1 mm (**a**–**e**), 50 µm (**a**_**1**_, **b**_**1**_, **d**_**1**_, **d**_**2**_)

CaBPs are molecules whose structures show significant evolutionary conservation (Andressen et al. 1993). Neuronal subtypes of select brain areas typically retain their CaBP expression profiles across vertebrate species (Andressen et al. 1993; Gati et al. 2014). The brainstem harbours vegetative centres and nuclei, which share similarity, or even identity, in both lower and higher order vertebrates. We selected the domestic chick (*Gallus domesticus*) as a precocial avian species with immediate maturity after hatching to verify our hypothesis that secretagogin has an evolutionary preserved neuron-specific distribution in the vertebrate brainstem. Thus, the vagal nucleus (Fig. 7a, a_1_), the ventrolateral medulla (Fig. 7b, b_1_), the vestibular nuclei and the locus coeruleus (Fig. 7c), midbrain nuclei like the ventral tegmental area and periaqueductal grey (Fig. 7d–d_2_) were typical secretagogin-expressing foci. Of note, we found secretagogin^+^ neurons in areas which cannot be directly correlated to mammalian brain structures. Thus, the supraspinal nucleus—innervating the upper neck muscles, but being separate from the nucleus where the accessory nerve arises (Wild 1981)—also contained immunoreactive cells (not shown) and smaller islets of secretagogin^+^ neurons were identified in different subdivisions of the reticular formation, especially in the midbrain (Fig. 7e). In conclusion, secretagogin is an evolutionarily conserved protein in many vertebrate brainstem nuclei.

### Secretagogin expression is reduced in noradrenergic brainstem nuclei in Alzheimer’s disease

**Fig. 8:**
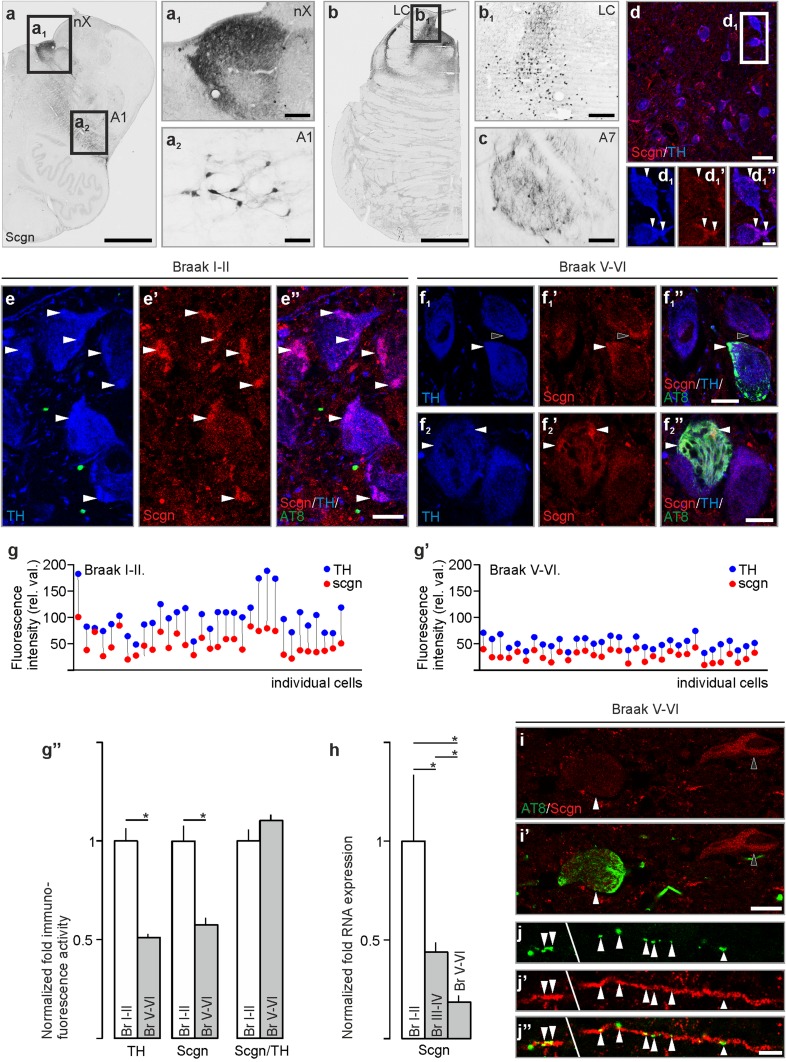
Secretagogin expression in the human brainstem is compromised in Alzheimer’s disease. **a**–**a**_**2**_ Secretagogin^+^ neurons of the medulla oblongate populated the dorsal nucleus of vagus (**a**_**1**_), captured from the immediate consecutive section after the section shown in **a** and the A1 field (**a**_**2**_). **b**, **b**_**1**_ In the pons, secretagogin^+^ neurons typically occurred in the locus coeruleus. **c** Secretagogin^+^ neurons in the A7 field. **d**–**d**_**1**_**’’** TH^+^ neurons of the human locus coeruleus expressed secretagogin. Secretagogin immunoreactivity concentrated along the cell membrane and most typically where dendrites emanated from the soma (arrowheads in **d**–**d**_**1**_**’’**). **e**–**e’’** Secretagogin was abundantly expressed in TH^+^ locus coeruleus neurons in individuals without histopathological signs of Alzheimer’s disease (Braak I–II) (arrowheads in **e**–**e’’** point to secretagogin-immunoreactive *loci* in TH^+^ soma. **f**_**1**_–**f**_**2**_**’’** In subjects with severe Alzheimer’s disease (Braak V–VI), secretagogin^+^ subcellular domains in TH^+^ somata were significantly reduced and often overlapped with the accumulation foci of AT8^+^ hyperphosphorylated tau (arrowheads in **f**_**1**_–**f**_**2**_**’’**, black arrowhead in **f**_**1**_–**f**_**1**_**’’** points to a secretagogin^+^/AT8^−^ domain). **g**, **g’** TH and secretagogin immunofluorescence showed a paralleled reduction in individual locus coeruleus neurons in Braak V–VI vs. Braak I–II subjects. **g’’** In average, TH-immunoreactivity decreased in locus coeruleus somata in severe Alzheimer’s disease which was paralleled with a loss in secretagogin immunoreactivity. **h** Secretagogin mRNA expression decreased significantly with the progress of Alzheimer’s disease in locus coeruleus micropunches. **i**–**i** Complementary distribution of AT8 and secretagogin immunoreactivity of locus coeruleus neurons in severe Alzheimer’s disease. **j**–**j’’** Initial, discontinuous accumulation of AT8^+^ tau protein (arrowheads) in secretagogin^+^ neuronal process in the locus coeruleus of a late-stage Alzheimer’s disease subject. **e**_**1**_, **f**_**1**_, **h***p* < 0.05, Student’s *t* test. *Scgn* secretagogin, *TH* tyrosine hydroxylase. Scale bars 500 µm (**a**, **b**), 100 µm (**a**_**1**_, **b**_**1**_, **c**), 25 µm (**a**_**2**_), 15 µm (**d**), 5 µm (**d**_**1**_**’’**, **e**–**e’’**, **i**–**i’**, **f**_**1**_–**f**_**2**_**’’**), 500 µm (**a**, **b**), 3 µm (**j’’**). Images **a**, **b**, **c** and **d**–**d**_**1**_**’’** were reproduced with permission from EMBO: Alpár et al., Hypothalamic CNTF volume transmission shapes cortical noradrenergic excitability upon acute stress. EMBO J. 2018 Nov 2;37(21). pii: e100087

Immersion fixation of blocked tissue, the routine processing procedure, and post-mortem delay adversely impact epitope detection by immunohistochemistry in human brain tissue. We overcame this problem by applying vascular perfusion via the internal carotid and vertebral arteries to preserve tissue integrity. Similarly to laboratory rodents, we found secretagogin^+^ neurons in the noradrenergic axis of the human brainstem (Fig. 8a–c), including the locus coeruleus (Fig. 8b, b_1_), and A1 (Fig. 8a_2_) and A7 (Fig. 8c) nuclei. TH^+^ locus coeruleus neurons co-expressed secretagogin, with subcellular *foci* (patches) restricted to peripheral intrasomatic domains and neuronal processes, frequently apposing the cell membrane (Fig. 8d–d_1_’’). In addition, we identified secretagogin^+^ neurons in the dorsal nucleus of vagus of the human brain (Fig. 8a, a_1_), similarly to what we found in rat (Fig. 4d_1_’) and mouse (Fig. 6c_1_).

We hypothesized that neurodegenerative stimuli may be harmful for secretagogin expression in LC neurons. Alternatively, secretagogin could spare these cells from neurodegeneration. Secretagogin^+^ hippocampal or olfactory neurons are preserved in neurodegenerative disorders, like Alzheimer’s disease (Attems et al. 2008, 2012a), supporting the latter hypothesis. However, it has been proposed that TH^+^ locus coeruleus neurons are most sensitive and compromised already in the early phase in Alzheimer’s disease (Tomlinson et al. 1981): tau pathology appears earliest in locus coeruleus noradrenaline neurons (Attems et al. 2012b). Monitoring secretagogin expression at the transcription (RNA) level, we found that it robustly decreased already in Braak III–IV stage subjects and further diminished in severe Alzheimer’s disease (Fig. 8h). In severe Alzheimer’s disease subjects (Braak V–VI), we identified a parallel decrease in TH (relative fluorescence intensity values: 103.59 ± 5.82 in Braak I–II vs. 52.66 ± 1.55 in Braak V–VI) and secretagogin (relative fluorescence intensity values: 47.13 ± 0.03 in Braak I–II vs. 27.42 ± 1.06 in Braak V–VI) immunoreactivity (Fig. 8e–g’’). In individuals with advanced stage of Alzheimer’s disease-related pathology (Braak V–VI), neurons with abundant cytoplasmic secretagogin immunoreactivity were very rare and often lacked AT8-immunoreactivity (hyperphosphorylated tau) (Fig. 8i, i’). Typically, secretagogin expression within TH^+^ neurons, if detectable, was restricted to very small intracellular compartments. These foci often overlapped with the AT8^+^ microdomains where tau hyperphosphorylation started to develop within the neuron (Fig. 8f_1_–f_1_’’, but see also Fig. 8f_2_–f_2_’’). Secretagogin expression was more evident in neuronal processes which occasionally showed multiple islets of AT8^+^ profiles (Fig. 8j–j’’). In conclusion, secretagogin expression decreased with the progression of Alzheimer’s disease, with residual immunoreactivity in degenerating intracellular microdomains.

## Discussion

The present study underlines the usefulness of secretagogin as a novel neurochemical marker to distinguish subsets of neurons in the vertebrate brain. Detected already during early ontogeny, secretagogin in adults was then expressed in important relay centres in the lower brain stem, notably in several of the noradrenaline groups, with the A6 locus coeruleus being of particular interest. Moreover, secretagogin expression in the locus coeruleus from Alzheimer’s disease subjects paralleled TH loss and was associated with initial, aberrant tau phosphorylation.

### Secretagogin is expressed early during ontogenesis and persists throughout adulthood in locus coeruleus neurons

CaBPs appear at different time points and last for different intervals during brainstem development. Calretinin, calbindin and parvalbumin are sequentially expressed in the rat auditory brainstem during development, correlating with definite developmental stages (Lohmann and Friauf 1996). The postnatal development of rat vestibular nuclei and their cerebellar projections could be mapped by the specific spatiotemporal appearance of the same CaBPs (Puyal et al. 2002). Notably, the early onset of the expression of these CaBPs is no guarantee for their ongoing presence through adulthood. For example, even if most neurons, which express calbindin D28k during development preserve their phenotype (Enderlin et al. 1987), this is not the case in the superior olivary complex, where calbindin D28k expression is transient and may reflect critical periods in the control of calcium homoeostasis (Friauf 1993). Calretinin’s transient expression during ontogenesis is even more pronounced, in mammals (Lohmann and Friauf 1996), fish (Porteros et al. 1998) and avian species (Bastianelli and Pochet 1993). Parvalbumin, in turn, may appear late (in fact postnatal) during forebrain development (Lohmann and Friauf 1996) and is induced by specific afferent stimuli (Barker and Dreher 1998; Manns and Gunturkun 2003; McHaffie et al. 2001). We here show that secretagogin appears early in mouse brainstem development and shows ongoing expression in mouse brainstem noradrenergic cells, notably of the locus coeruleus neurons. Speculatively, secretagogin may regulate calcium-dependent mechanisms or TH synthesis in noradrenergic neurons, which are critical not only in adulthood but already in embryonic life.

### Secretagogin expression in relay, vegetative and stress centres of the vertebrate brainstem

Secretagogin was expressed in nuclei with different functions. We highlight that secretagogin^+^ neurons (i) populate the brainstem nuclei which serve as major vegetative command centres, such as the parabrachial or the solitary tract nucleus, (ii) occur in critical relay stations in the pathway of special senses (vestibular and visual), (iii) outline the brainstem noradrenaline axis and (iv) that this expression pattern has been preserved in phylogenesis, from avian species to humans. At the same time, secretagogin was expressed neither in serotonin^+^ raphe neurons nor in TH^+^ neurons in the ventral tegmentum, i.e. dopamine neurons, and we could reconfirm (data not shown) that cholinergic neurons at the midbrain–hindbrain border, including the pedunculopontine nucleus (Ch5), the laterodorsal tegmental nucleus (Ch6) and the parabigeminal nucleus (Ch8), remained immunonegative for secretagogin (Kosaka and Kosaka 2018).

CaBPs are widely used as neurochemical markers to distinguish neuronal pools (Andressen et al. 1993; Celio 1990; Jacobowitz and Winsky 1991), with their exact intracellular function, however, remaining contradictory (Freund et al. 1990) or largely unrevealed. In general terms, Ca^2+^-sensor proteins, including secretagogin (Wagner et al. 2000), undergo a conformational change upon cell activation (Rogstam et al. 2007) to trigger intracellular signalling events (Gartner et al. 2007; Hanics et al. 2017; Malenczyk et al. 2017). Thus, secretagogin can be critical to regulate vegetative functions and sensory processing at the brainstem level based on their specific expression in the above nuclei. To unravel secretagogin’s physiological role in brainstem nuclei, future experiments could investigate the vegetative, vestibular and visual systems. We would definitely consider loss-of-function and activity-dependence experiments which impact the vegetative control at the major supraspinal level (solitary tract nucleus), control of balance and relevant motor skills as well as visual functions controlled at the midbrain level (e.g. smooth pursuit). Finally, whilst we recently explored secretagogin’s role in a mechanism converting hypothalamic activation into long-lasting cortical excitability following acute stress (Alpar et al. 2018), secretagogin’s function in shaping the ascending and descending reticulo-activating system is a promising field for future works.

### Is secretagogin involved in somato-dendritic release of neuropeptides?

It has been shown that secretagogin interacts with proteins implicated in i.a. docking of release vesicles (Bauer et al. 2011; Romanov et al. 2015). In fact, there is evidence that secretagogin is localized in corticotropin-releasing hormone (CRH) axon terminals at the median eminence and modulates release of this neuropeptide at this site (Romanov et al. 2015). The present results show that secretagogin in human locus coeruleus neuron cell bodies and dendrites has a patchy distribution not rarely close to the cell membrane. We have proposed that the neuropeptide galanin is released from soma and dendrites of rat (Vila-Porcile et al. 2009) and human (Barde et al. 2016) locus coeruleus neurons under stressful conditions. This type of release has been shown for oxytocin and vasopressin to occur in the hypothalamic magnocellular neurons (Ludwig and Leng 2006). It may be relevant to analyse if secretagogin in the locus coeruleus neurons is involved in the regulation of galanin release, since this peptide system is involved in mood disorders (Hokfelt et al. 2018; Holmes and Picciotto 2006; Kuteeva et al. 2010; Lu et al. 2007).

### Secretagogin expression decreases in Alzheimer’s disease

Ca^2+^-sensor proteins work in an activity-dependent manner to control downstream signalling. Cell activity and excitability are reduced in neurodegenerative diseases, including Alzheimer’s disease (de Haan et al. 2017). We found that secretagogin expression significantly decreased with disease progression, paralleling TH loss typical already in the early phase of Alzheimer’s disease (Braak and Del Tredici 2012; Chan-Palay and Asan 1989; German et al. 1992; Kelly et al. 2017), which may reflect impaired cell function and activity.

Previous studies suggested that secretagogin is neuroprotective in hippocampal pyramidal cells in Alzheimer’s disease (Attems et al. 2008) and secretagogin expression has recently been shown to parallel disease progression: P301L tau transgenic mice showed reduced secretagogin expression in hippocampal neurons (Attems et al. 2011). Whilst we found that structural malformations can be independent of secretagogin loss (neurons with robustly reduced or not detectable secretagogin expression did not necessarily contain AT8^+^ hyperphosphorylated tau), small intracellular domains with residual secretagogin expression were identified as foci with initial tau aberrancies and islet-like multiple accumulation of AT8^+^ tau repeatedly occurred in the secretagogin^+^ processes. This may implicate that secretagogin-expressing domains can resist structural degradation which refers to its previously suggested role in neuroprotection (Attems et al. 2008). Accumulating evidence suggest that a deregulation of calcium signalling may play a major role in Alzheimer’s disease progression; CaBPS such as parvalbumin, calbindin and calretinin are upregulated in the hippocampus of 3-month-old APPswe/PS1dE9 transgenic mice, possibly to control cellular homoeostasis and synaptic plasticity, but losing cellular capacity to pathophysiological processes by the age of 12 months (Verdaguer et al. 2015). Activity-dependent translocation of synaptonuclear factors from synapses to the nucleus is regulated by calmodulin-dependent mechanisms; altered synapse-to-nucleus signalling may lead to neurodegenerative and neuropsychiatric diseases (Parra-Damas and Saura 2019). Ca^2+^/calmodulin-dependent protein kinase kinase 2 controls important neuronal processes and its loss leads to aberrant transferrin phosphorylation and trafficking which makes it a potential biomarker for Alzheimer’s disease (Sabbir 2018). CaBPs-mediated mechanisms are also important in glial cells to resist neurological disorders. Calcium dysregulation triggers astrocyte activation which leads to neuroinflammation, release of synaptotoxic factors and loss of glutamate regulation which can finally culminate in neurodegeneration (Sompol and Norris 2018). Dysregulation of calcineurin signalling pathways in activated astrocytes and its interaction with the nuclear factor of activated T cells (NFATs) couple vascular pathology to neurodegeneration and cognitive loss (Kraner and Norris 2018). Calcium sensor proteins have been previously implicated in neuroprotection: dopamine neurons in the substantia nigra use calretinin to confer oestrogen’s effect to prevent cell loss (Yi et al. 2016) and calretinin cooperates with the NCX1 exchanger to resist neurodegeneration (Boscia et al. 2016).

We suggest that secretagogin’s involvement in Alzheimer’s disease is (i) due to its calcium sensor rather than to its calcium buffer property. Secretagogin has been shown to regulate diverse cellular mechanisms and functions, including migration or hormonal release, in distinct brain neuronal populations (Alpar et al. 2018; Hanics et al. 2017; Romanov et al. 2015). All these functions seemed to be linked to secretagogin’s calcium sensor property. Second (ii), secretagogin’s involvement in Alzheimer’s disease is likely exerted by affecting noradrenergic cell function, hence modulating noradrenergic cortical input. In conclusion, secretagogin as a calcium sensor may act neuroprotectively by regulating downstream machineries in noradrenergic cells.

## Materials and methods

### Animals, surgery and ethical approval of experimental studies

Eleven 6-week-old male rats (Wistar), three 12-week-old male wild-type mice (C57BL/6), a total of six embryos from three pregnant C57BL/6 mice, three 12-week-old male EGFP–secretagogin transgenic mice and three 14-day-old chicks (*Gallus domesticus*) were used. Food and water were available ad libitum and animals were kept under standard housing condition and using a 12/12 light/dark cycle. Experimental procedures, including stereotaxic injections and transcardial perfusion, were approved by the Ethical Review Board of the Semmelweis University and conformed to the European Convention for the Protection of Vertebrate Animals used for experimental and other scientific purposes (Protocols: ETS No. 170, ETS No.123, Tierversuchgesetz 2012, BGBI, Nr. 114/2012). Animals during surgeries and transcardial perfusions were anesthetized intramuscularly (i.m.) or intraperitoneally (i.p.) with a mixture of ketamine (50 mg/kg b wt) and xylazine (4 mg/kg b wt). After surgery, brains were perfusion fixed transcardially with 4% paraformaldehyde (PFA) in 0.1 M phosphate buffer (0.1 M PB).

### Production of the SB-BAC-SCGN-EGFP transgenic mouse line

To generate transgenic mice expressing the EGFP (enhanced green fluorescent protein) under the control of the SCGN promoter, we used a BAC (Bacterial Artificial Chromosome) engineering technology (Lee et al. 2001). The B6Ng01-268C03 (RIKEN BioResource Research Center) BAC clone was chosen, which contained the whole SCGN gene and downstream of it another harbouring gene (Hist1h2ba) which was removed later. In the BAC modification casette, the EGFP cDNA was inserted between the recombination arms and its ATG site was fused into the SCGN’s gene ATG site. Following the EGFP cDNA, a WPRE (woodchuck hepatitis virus (WHP) posttranscriptional regulatory element), an hGH-PA (human growth hormone polyadenylation signal) and a neomycin selection marker flanked by flippase recognition sites were inserted into the construct. Recombination was carried out as described by Lee et al. (2001). After the insertion of the EGFP and other components into the BAC, the neomycin marker was removed in the host cells by a flippase enzyme. This was followed by a second recombineering-based BAC modification step in which ~ 40 kb downstream region from the SCGN gene was removed, which contained the neighbour gene. In the same step, we inserted both ITR (inverted terminal repeat) sequences recognized by the Sleeping Beauty transposase to combine the BAC transgenesis with a transposon-based system for increased integration efficiency (Rostovskaya et al. 2012). Transgenic mice were derived by pronuclear microinjection of the SB-BAC-SCGN-EGFP BAC (circular, 1 ng/μl), Sleeping Beauty transposase mRNA (5 ng/μl) into C57BL/6Ntac fertilized eggs.

### Immunohistochemistry and imaging

Chromogenic or multiple immunofluorescence histochemistry with select combinations of primary antibodies (Table 1) was performed according to published protocols (Alpar et al. 2010; Lendvai et al. 2013). Free-floating sections (30 μm) were rinsed in phosphate buffer (PB, pH 7.4) and pre-treated with 0.3% Triton X-100 (in PB) for 1 h at 22–24 °C to enhance the penetration of antibodies. Non-specific immunoreactivity was suppressed by incubating our specimens in a cocktail of 5% normal donkey serum (NDS; Jackson), 2% bovine serum albumin (BSA; Sigma) and 0.3% Triton X-100 (Sigma) in PB for 1 h at 22–24 °C. Sections were exposed (16–72 h at 4 °C) to select combinations of primary antibodies (Table 1, (Alpar et al. 2004; Hanics et al. 2017; Lendvai et al. 2013; Mulder et al. 2010; Romanov et al. 2015)) diluted in PB to which 0.1% NDS and 0.3% Triton X-100 had been added. After extensive rinsing in PB, the sections were processed using chromogenic or immunofluorescence detection. In single-labelling experiments, the sections were exposed to biotinylated anti-rabbit IgG raised in donkey (1:1,000 [Jackson], 2 h at 22–24 °C) followed by pre-formed avidin–biotin complexes also incorporating horseradish peroxidase for 1 h at 22–24 °C. Immunosignals were visualized by 3,3′-diaminobenzidine (Sigma, 0.025%) as chromogen intensified with Ni-ammonium sulphate (0.05%, Merck) in the presence of 0.001% H_2_O_2_ as substrate (dissolved in 0.05 M Tris buffer, pH 8.0). In multiple immunofluorescence labelling experiments, immunoreactivities were revealed by carbocyanine (Cy) 2, 3 or 5-tagged secondary antibodies raised in donkey (1:200 [Jackson], 2 h at 22–24 °C). Glass-mounted sections were coverslipped with glycerol/gelatin (GG-1; Sigma).

**Table 1:** List of markers used for immunolabelling

Marker	Source	Host	IH dilution	References
Calbindin	Synaptic systems	guinea pig, pc^2^	1:1000	Hanics et al. (2017)
Calretinin	Synaptic systems	guinea pig, pc^2^	1:1000	Hanics et al. (2017)
Parvalbumin	Millipore	Mouse, mc^1^ (AT8)	1:1000	Lendvai et al. (2013)
PHF tau	Pierce	Mouse, mc^1^ (AT8)	1:1000	Lendvai et al. (2013)
Secretagogin	L. Wagner	Rabbit, pc^2^	1:12,000	Romanov et al. (2015)
Secretagogin	Cell signaling	Rabbit, pc^2^	1:1000	This study
Secretagogin	R&D systems	Goat, pc^2^	1:100	Mulder et al. (2010)
Tyrosine hydroxylase	MERCK/Millipore	Rabbit, pc^2^	1:1000	Alpar et al. (2004)

^1^Monoclonal antibody

^2^Polyclonal antibody

The results of chromogenic stainings were captured on an Olympus BX-51 microscope at 10×, 20× and 40× primary magnification. Sections processed for multiple immunofluorescence histochemistry were inspected and images acquired on a 780LSM confocal laser-scanning microscope (Zeiss) with optical zoom ranging from 1× to 3× at 63× primary magnification (Plan-Apochromat 63×/1.40), and pinhole settings limiting signal detection to 0.5–0.7 μm “optical thickness”. Emission spectra for each dye were limited as follows: Cy2 (505–530 nm), Cy3 (560–610 nm), and Cy5 (650–720 nm). Multi-panel figures were assembled in CorelDraw X5 (Corel Corp.).

We used the histogram toolbox of the ZEN software (ZEISS) to measure immunofluorescence in the locus coeruleus neurons of Braak I–II (*n* = 3) and Braak V–VI (*n* = 3) subjects. Images from all sections (*n* = 3 per subject) were captured using identical settings. Somata (*n* = 11 per section) were demarcated and their immunofluorescence, reflecting their TH and secretagogin immunoreactivity, were automatically measured and their quotient calculated.

To measure the overlap of secretagogin–EGFP expression and secretagogin immunoreactivity, we developed secretagogin immunoreactivity using the anti-secretagogin antibody raised in rabbits on sections made from secretagogin–EGFP mice brainstem. Analyses were carried out on images captured at 20× primary magnification. EGFP-expressing and immunoreactive somata were labelled with different markers in different layers (channels) using Photoshop which allowed co-localization of the two markers. In all three animals and for every nucleus, EGFP expression/immunoreactivity of somata were identified and marked in a minimum of three corresponding sections which were consecutive serial 30 μm-thick-sections of a four series-section pool. Labelled cells were counted in the whole nucleus on the section. The occurrence of secretagogin–EGFP expression or secretagogin immunoreactivity in double-labelled somata was calculated and expressed as average ± SEM. Co-localization coefficients between secretagogin, parvalbumin, calbindin and calretinin were measured and calculated along the same principle, including animal number and the quantitative attributes of image analysis. We emphasize that this approach was an investigative method and did not aim to provide accurate results regarding cell numbers/proportions.

### Human tissue preparation and immunohistochemistry

We applied direct perfusion via the internal carotid and vertebral arteries, which facilitated the preservation of tissue integrity relative to alternative fixation methods. Human brains (*n *= 2, gender and age: female/83 years and male/79 years, with a clinical history lacking neurodegenerative disease, ethical approval: TUKEB 84/2014, Hungary) were first perfused with physiological saline, followed by a fixative containing 2% PFA and 0.1% glutaraldehyde in 0.1 M Tris-buffered saline (TBS, pH 7.4) 7 h or 11 h after death. The removal and subsequent preparation of human tissues were in accordance with the relevant ethical guidelines of Semmelweis University (1998, Budapest, Hungary). Blocks from the medulla oblongata and pons were dissected out and post-fixed in 2% PFA in TBS for 72, followed by immersion in cryoprotective 30% sucrose in 0.1 M PB (pH 7.4) overnight. Coronal sections (50 μm) were cut on a cryostat microtome and processed for immunohistochemistry. Free-floating sections were rinsed in PB (pH 7.4) and pre-treated with 0.3% Triton X-100 (in PB) for 1 h at 22–24 °C to enhance the penetration of antibodies. Non-specific immunoreactivity was suppressed by incubating our specimens in a cocktail of 5% NDS (Jackson), 10% BSA (Sigma) and 0.3% Triton X-100 (Sigma) in PB for 1 h at 22–24 °C. Sections were exposed for up to 72 h (at 4 °C) to the cocktail of primary antibodies (Table 1) diluted in PB to which 0.1% NDS and 0.3% Triton X-100 had been added. After extensive rinsing in PB, the immunoreactivities were revealed by chromogenic staining (as above) or by Cy2, 3 or 5-tagged secondary antibodies raised in donkey (1:200 [Jackson], 2 h at 22–24 °C). Lipofuscin autofluorescence was quenched by applying Sudan Black-B [1%, dissolved in 70% ethanol (Schnell et al. 1999)]. Glass-mounted sections were coverslipped with Aquamount embedding medium (Dako). Sections were inspected and images acquired on a 710LSM confocal laser-scanning microscope (Zeiss) at 10× or 40× primary magnification and pinhole settings limiting signal detection to 0.5–0.7 μm. Emission spectra for each dye were limited as follows: Cy2/505–530 nm, Cy3/560–610 nm, and Cy5/650–720 nm. Multi-panel figures were assembled in CorelDraw X7 (Corel Corp.).

Samples from patients with Alzheimer’s disease and age-matched controls (without clinical signs of neuropsychiatric disease) were acquired at the Brain Bank of the Institute of Neurology, Medical University of Vienna, Austria, and from the Human Brain Bank of Semmelweis University, Budapest, Hungary (a total of 34 samples were used, for details see Table 2). For immunohistochemistry, pontine blocks including the locus coeruleus were immersion fixed in 4% PFA in PB for 2–3 weeks at room temperature. These cases had previously been characterized for Alzheimer’s disease-related pathologies (amyloid β (Aβ) plaque burden, intracellular tau accumulation) and other neurodegenerative conditions had been excluded. Tissues were obtained and used compliant with the Declaration of Helsinki and following institutional guidelines. The study was performed in the course of an approved study by the Ethical Committee of the Medical University of Vienna (No.1454/2018). All patient material was coded to ensure anonymity throughout tissue processing. For real-time quantitative PCR experiments, locus coeruleus micropunches from human samples were homogenized in isolating buffer (Qiagen). RNA was extracted using the RNeasy mini kit (Qiagen) with a DNase I step performed to eliminate traces of genomic DNA30 and reverse transcribed using a high-capacity cDNA reverse transcription kit (Applied Biosystems). Reactions were performed after an initial denaturation at 95 °C for 10 min, followed by 40 cycles of 95 °C for 15 s denaturation, annealing and extension at calculated temperatures (60 s) and a dissociation stage (from 60 to 95 °C with 0.5 °C steps for 10 s each; CFX96, Bio-Rad), with primer pairs amplifying short fragments for the secretagogin gene (forward: 5′-CTGTTAGATGGCTCTGCCTGTC-3′, reverse: 5′-GTTACAGGATTGCCATGAATGC-3′). Samples without template or reverse transcriptase served as negative controls. Expression levels were normalized to the housekeeping gene encoding glyceraldehyde-3-phosphate dehydrogenase (GAPDH, forward: 5′–AACTTTGGCATTGTGGAAGG–3′, reverse: 5′ACACATTGGGGGTAGGAACA3′) obtained for every sample in parallel assays. Quantitative (q)PCR was performed on a Bio-Rad CFX 96 thermal cycler. Samples were run in triplicate to avoid processing-related deviations.

**Table 2:** Demography and use of human subjects

Case ID	Stage	Age (year)	Gender	Analysis
#130-03	Braak I–II	78	Male	IHC
#84-03	Braak I–II	77	Male	IHC
#129-03	Braak I–II	77	Female	IHC
#15-05	Braak I–II	78	Female	IHC
#126-05	Braak I–II	80	Female	IHC
#14-05	Braak III–IV	78	Male	IHC
#156-03	Braak III–IV	77	Male	IHC
#131-05	Braak III–IV	79	Female	IHC
#58-06	Braak III–IV	80	Female	IHC
#100-07	Braak III–IV	81	Male	IHC
#9-10	Braak V–VI	84	Male	IHC
#124-07	Braak V–VI	81	Female	IHC
#88-05	Braak V–VI	78	Male	IHC
#103-06	Braak V–VI	81	Female	IHC
#107-10	Braak V–VI	85	Female	IHC
#1	Braak I	63	Male	qPCR
#90	Braak I	78	Female	qPCR
#98	Braak I	74	Male	qPCR
#164	Braak I	85	Male	qPCR
#165	Braak I	88	Female	qPCR
#187	Braak I	62	Male	qPCR
#189	Braak I	79	Female	qPCR
#220	Braak I–II	63	Male	qPCR
#90	Braak I	78	Female	qPCR
#98	Braak I	74	Male	qPCR
#164	Braak I	85	Male	qPCR
#165	Braak I	88	Female	qPCR
#187	Braak I	62	Male	qPCR
#189	Braak I	79	Female	qPCR
#220	Braak I–II	63	Male	qPCR
#94	Braak III–IV	65	Male	qPCR
#185	Braak III–IV	80	Male	qPCR
#196	Braak III–IV	78	Female	qPCR
#197	Braak III–IV	64	Male	qPCR
#222	Braak III–IV	80	Male	qPCR
#163	Braak V–VI	81	Female	qPCR
#177	Braak V–VI	89	Female	qPCR
#188	Braak V–VI	86	Female	qPCR
#192	Braak V–VI	81	Male	qPCR
#195	Braak V–VI	83	Male	qPCR
#201	Braak V–VI	74	Male	qPCR
#185	Braak III–IV	80	Male	qPCR
#196	Braak III–IV	78	Female	qPCR
#197	Braak III–IV	64	Male	qPCR
#222	Braak III–IV	80	Male	qPCR
#163	Braak V–VI	81	Female	qPCR
#177	Braak V–VI	89	Female	qPCR
#188	Braak V–VI	86	Female	qPCR
#192	Braak V–VI	81	Male	qPCR
#195	Braak V–VI	83	Male	qPCR
#201	Braak V–VI	74	Male	qPCR

### Whole mounts

Neural tissue explants were isolated from E8.5 and E11.5 mouse embryos from pregnant mice (*n* = 4 in total) with a modified technique described elsewhere (Echevarria et al. 2001). Briefly, neural tubes were opened by cutting the roof plate on ice-cold PBS. Once flattened as an open book, they were placed on Millicell cell culture plate inserts (Millipore, pore size 0.4 μm) and cultured for 3 days as on Neurobasal medium penicillin (100 U/mL), and streptomycin (100 μg/mL) (all from Invitrogen) in an incubator at 37 °C, with 5% CO_2_ and 95% humidity. The neural tube explants were subsequently removed from the membranes, fixed in PFA4% and immunostained as described above.

### Statistical analysis

Data were analysed using Statistical Package for the Social Sciences (version 17.0, SPSS Inc.). Immunofluorescence intensities were evaluated using Student’s *t* test (on independent samples, *p < 0.05). qPCR data were analysed using one-way ANOVA with Tukey’s post hoc tests. Data were expressed as mean ± SEM. A *p* value of < 0.05 was considered statistically significant.

## Electronic supplementary material

Below is the link to the electronic supplementary material.

Supplementary material 1 (DOCX 1132 kb)
