# Supplementary material for: Secretagogin expression in the vertebrate brainstem with focus on the noradrenergic system and implications for Alzheimer’s disease
Source: Brain Struct Funct. 2019 May 29;224(6):2061–78. doi: 10.1007/s00429-019-01886-w (PMC6591208; doi:10.1007/s00429-019-01886-w)
Supplement: Supplementary file 1 — Supplementary material 1 (DOCX 1132 kb) [file 429_2019_1886_MOESM1_ESM.docx]

Electronic Supplementary Material to:

**Secretagogin expression in the vertebrate brainstem – a focus on the noradrenergic system and implications for Alzheimer’s disease** (P. Zahola *et al.,* Brain Structure and Function)

**Contents**

**Figure S1** Secretagogin^+^ neurons did not co-express TH and serotonin in the ventral tegmentum and median raphe, respectively.

**Figure S2** Colocalizations of and colocalization coefficients between secretagogin-EGFP and secretagogin-immunoreactivity in the Scgn-EGFP mouse brain

**Figure S3** Differences in secretagogin expression of rats and mice brain stem

**
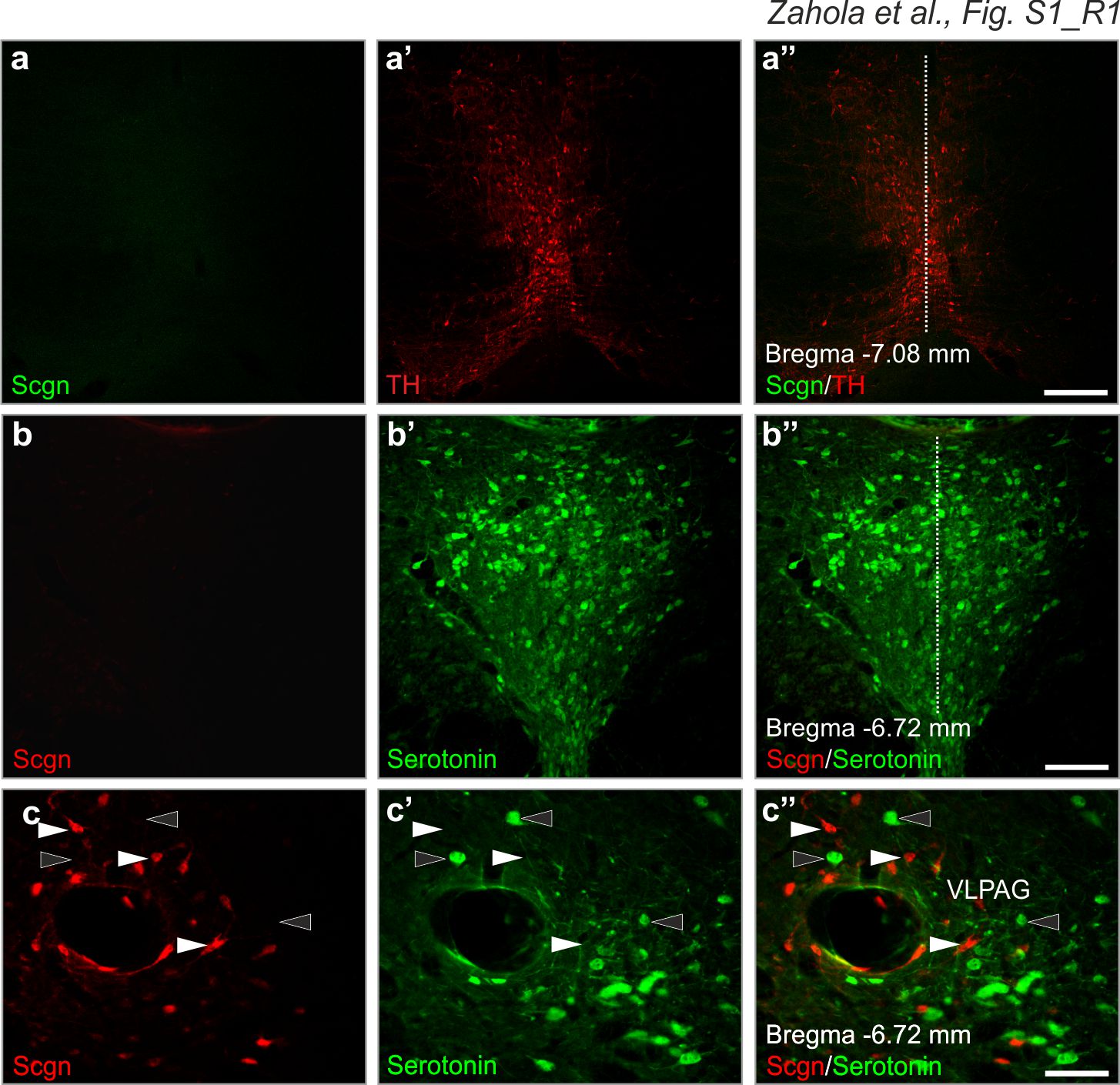
**

**Fig. S1 Secretagogin^+^ neurons did not co-express TH and serotonin in the ventral tegmentum and median raphe, respectively.** (a-a’’) TH^+^ neurons in the midbrain ventral tegmentum did not co-express secretagogin. (b-b’’) Serotonin^+^ neurons of the midbrain raphe remained immunonegative for secretagogin. (c-c’’) Dual immunolabeling showed a complementary distribution of secretagogin and serotonin in the ventrolateral part of the periaqueductal gray (*white arrowheads* point to secretagogin^+^/serotonin^-^ neurons, *black arrowheads* point to secretagogin^-^/serotonin^+^ neurons). Dashed lines in a’’ and b’’ indicate the midline.  *Abbreviation* Scgn secretagogin, VLPAG ventrolateral part of the periaqueductal gray. *Scale bars* 150 µm (a’’, c’’), 40 µm (b’’).


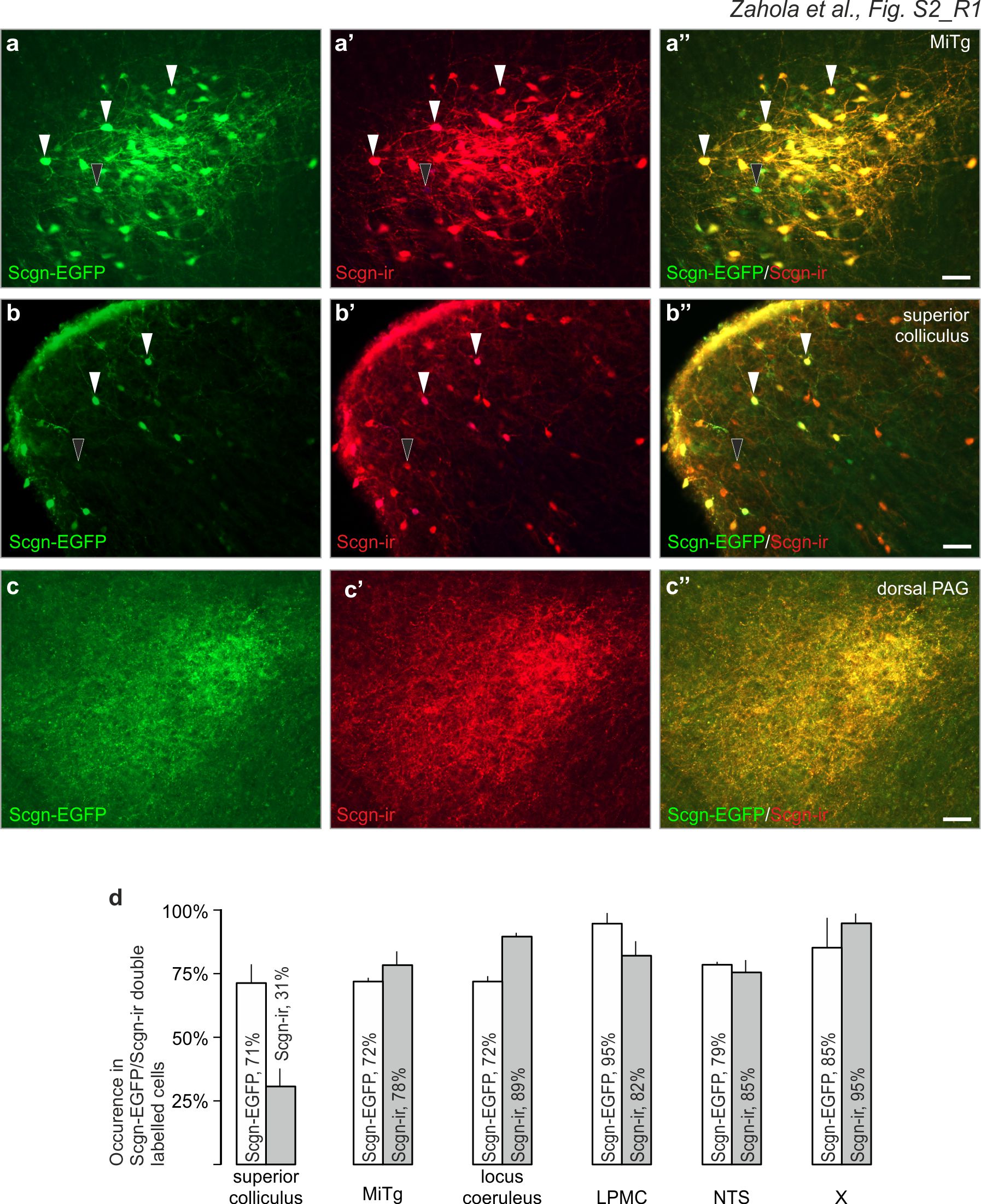


**Fig. S2 Colocalizations of and colocalization coefficients between secretagogin-EGFP and secretagogin-immunoreactivity in the Scgn-EGFP mouse brain.** (a-a’’) Colocalization of secretagogin-EGFP and secretagogin-immunoreactivity in the microcellular tegmental nucleus. *White arrowheads* point to secretagogin-EGFP^+^/secretagogin^-^immunopositive somata, *black arrowhead* points to a secretagogin-EGFP^+^/secretagogin-immunonegative cell body. (b-b’’) Colocalization of secretagogin-EGFP and secretagogin-immunoreactivity in the superior colliculus. *White arrowheads* point to secretagogin-EGFP^+^/secretagogin-immunopositive somata, *black arrowhead* points to a secretagogin-EGFP^+^/secretagogin-immunonegative cell body. (c-c’’) Colocalization of secretagogin-EGFP and secretagogin-immunoreactivity in fibres in the dorsolateral part of the periaqueductal gray. (d) Frequency of secretagogin-EGFP expression and secretagogin-immunoreactivity in secretagogin-EGFP^+^/secretagogin-immunopositive somata in mouse brainstem nuclei. *Abbreviation* LPMC lateral posterior thalamic nucleus mediocaudal part, MiTG microcellular tegmental nucleus, NTS solitary tract nucleus, PAG periaqueductal gray, X dorsal nucleus of vagus. *Scale bars* 300 µm.


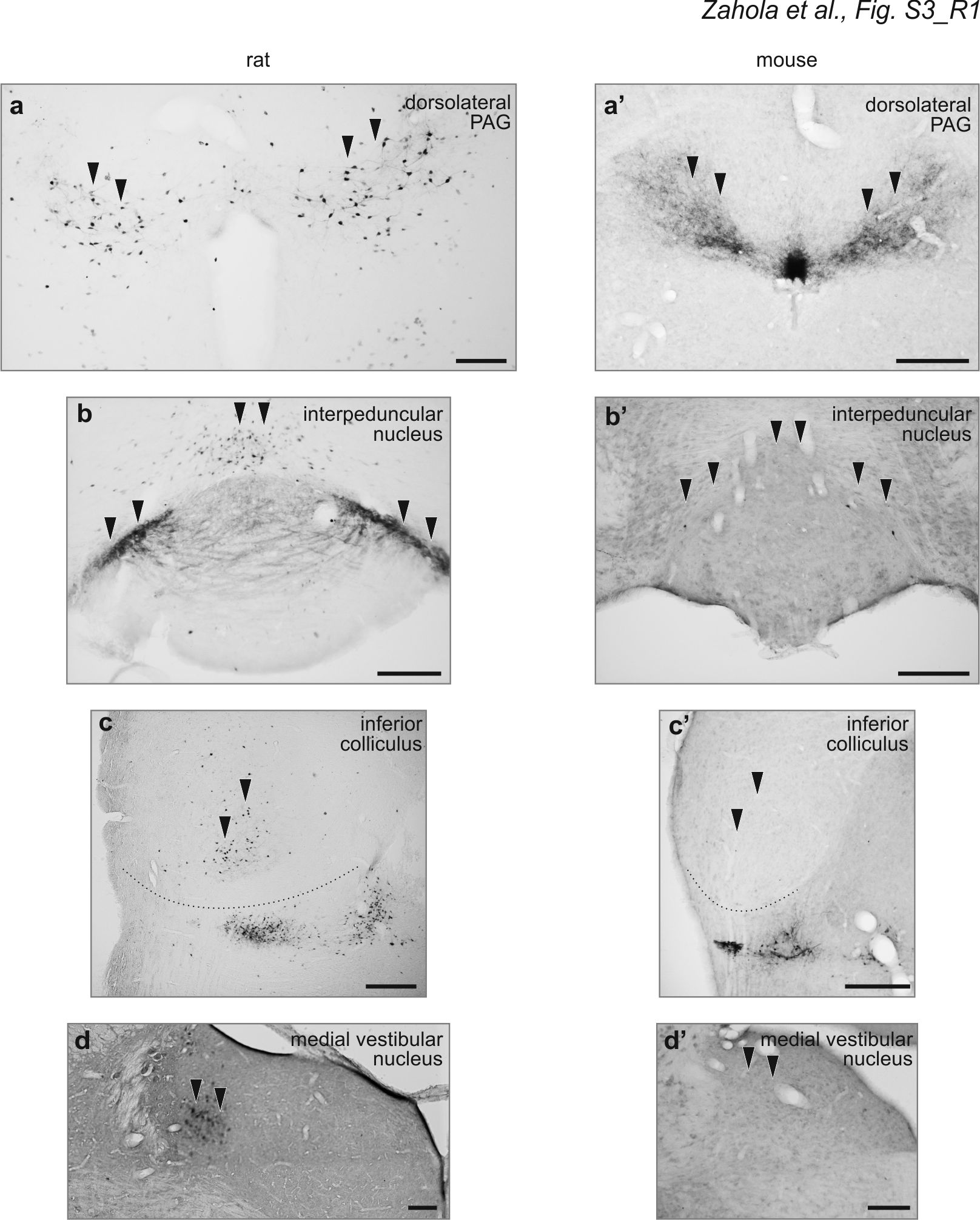


**Fig. S3 Differences in secretagogin expression of rats and mice brain stem.** (a, a’) The dorsolateral part of the periaqueductal gray contained immunoreactive somata but fibres and terminals in rats and mice brains, respectively. (b, b’) In rats, we detected secretagogin^+^ somata and somata/fibres in the dorsal and lateral parts of the interpeduncular nucleus, respectively, but we found no immunoreactivity in mice interpeduncular nucleus. (c, c’) Immunoreactive somata in the ventral part of the inferior colliculus in rats, but not in mice. Dashed curve indicates the bottom border of the inferior colliculus. (d, d’) Secretagogin^+^ somata in the medial vestibular nucleus in rats but not in mice brains. In all captures, *arrowheads* indicate the region of interest.  *Abbreviation* PAG periaqueductal gray. *Scale bars* 300 µm.

|  |  |  |  |
| --- | --- | --- | --- |
| **Table S1. Occurence of secretagogin-EGFP and secretagogin-immunoreactivity in double labelled cells in mouse brainstem nuclei.** | | | |
|  |  |  |  |
| **Region** | **EGFP in double labelled somata (%)** | **Immunoreactivity in double labelled somata (%)** |  |
| Superior colliculus | 71,3 ± 7,8 | 30,6 ± 7,3 |  |
| Microcellular tegmental nucleus | 70,2 ± 2,9 | 78,2 ± 5,5 |  |
| Locus coeruleus | 71,9 ± 2,3 | 89,3 ± 1,3 |  |
| Nucl. LPMC | 94,5 ± 4,5 | 81,8 ± 6,4 |  |
| Solitary tract nucleus (NTS) | 78,8 ± 0,2 | 75,4 ± 5,4 |  |
| Dorsal vagal nucleus (X) | 85,4 ± 11,2 | 95,0 ± 4,1 |  |
| Parabrachial nucleus | 91,2 ± 4,6 | 84,4 ± 7,9 |  |
|  |  |  |  |
| *n* = 3, average ± s.e.m. |  |  |  |
| Immunoreactivity was developed using the anti-secretagogin antibody raised in rabbit. | | |  |
|  |  |  |  |
| We acknowledge that the above proportions were calculated using an investigative method and not by stereology, thus, | | |  |
| it provides approximate instead of accurate results regarding cell numbers/proportions. | | |  |
